# Giant 8-cm coronary artery aneurysm: Surgical management

**DOI:** 10.1016/j.xjtc.2025.06.026

**Published:** 2025-07-18

**Authors:** Alexandra Murillo-Solera, Vaishnavi Karanam, Ryan Azarrafiy, Iverson E. Williams, Oscar Holmvard, Muhammad F. Umar, Thomas M. Beaver

**Affiliations:** a Division of Cardiovascular Surgery, Department of Surgery, University of Florida, Gainesville, Fla; b Department of Internal Medicine, AdventHealth Tampa, Tampa, Fla

**Keywords:** coronary artery aneurysm, giant coronary aneurysm, coronary artery bypass graft, cardiac surgery

## Abstract

We present the case of a 75-year-old man presenting asymptomatically with a large 8-cm right coronary artery aneurysm. The right coronary artery aneurysm was identified by cardiac catheterization and managed through coronary artery bypass grafting. The patient had an uneventful postoperative course and showed stable recovery at his follow-up visit.

**Figure:**
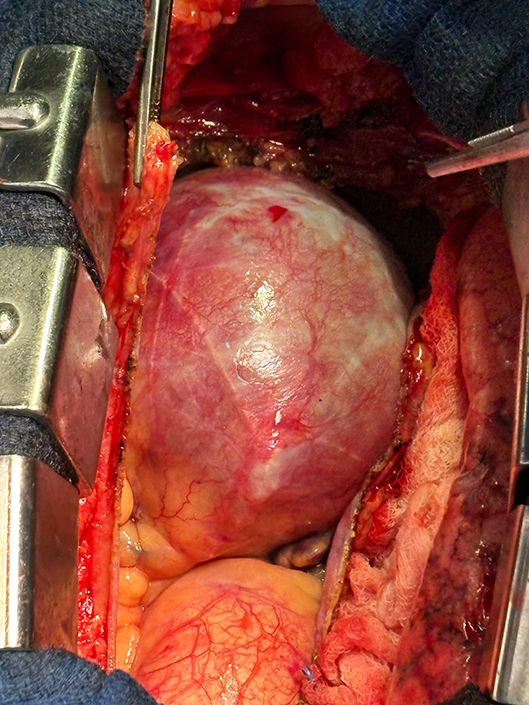
Large 8-cm right coronary artery aneurysm.

**Central Message**

In this case, we provide a clear, step-by-step approach to tackling intricate, high-risk right coronary artery aneurysm cases, which often present unique surgical challenges.

**Perspective**

We present the case of a 75-year-old man presenting asymptomatically with a large 8-cm RCAA. The RCAA was identified by cardiac catheterization and managed through coronary artery bypass grafting. The patient had an uneventful postoperative course and showed stable recovery at his follow-up visit.

A coronary artery aneurysm (CAA) is characterized by a localized enlargement of the coronary artery that exceeds 1.5-fold diameter of the adjacent normal segments.^1^ CAAs are a silent, progressive disorder that can be detected by coronary angiography.^1^ CAA is a rare disorder, detected in 0.3% to 4.9% of patients undergoing coronary angiography.^2^ It can lead to fatal complications such as rupture, thrombus formation, distal embolization, and compression of nearby cardiopulmonary structures.^1^ Atherosclerosis accounts for more than 90% of CAAs in adults.^2^ Other causes include Kawasaki disease, other vasculitis (such as Takayasu's arteritis and polyarteritis nodosa), lupus erythematosus, connective tissue disorders, congenital defects, infections (such as narcotic emboli, syphilis, and Lyme disease), trauma, dissection, cocaine abuse, and iatrogenic and idiopathic origins.^3^

We present the case of a 75-year-old man presenting asymptomatically with a large, 8-cm Right CAA (RCAA). The patient had a past medical history of hypertension, hyperlipidemia, coronary artery disease, type 2 diabetes, paroxysmal atrial fibrillation, chronic obstructive lung disease, transient ischemic attack, and obstructive sleep apnea. He had no prior surgical history. Although asymptomatic, abnormal electrocardiogram findings during a routine clinic visit prompted a stress test, which was positive and was followed by cardiac catheterization that identified a large RCAA.

Preoperative cardiac catheterization identified a large RCAA and revealed disease in the obtuse marginal artery (OMA), but no significant lesions in the left anterior descending artery. Preoperative computed tomography angiography showed an 8-cm mass, significantly compressing the right atrium and right ventricle, along with very calcified coronary arteries (Figure 1). Surgical intervention included coronary artery bypass grafting (CABG) with reverse saphenous vein grafts to the right coronary artery and to the OMA, along with an atrial appendage clip and pulmonary vein isolation for his paroxysmal atrial fibrillation. The operative technique is shown in Video 1.

**Figure 1:**
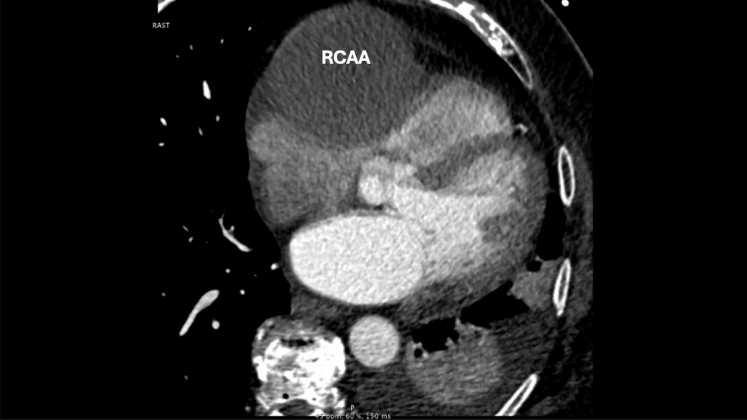
Computed tomography angiography demonstrating a right coronary artery aneurysm (RCAA).

Intraoperatively, the chest was entered through a median sternotomy. Upon opening the chest and dissecting the pericardium, a large mass became clearly visible (Figure 2). Aortic and right atrial cannulation was completed, a crossclamp was placed, and antegrade cardioplegia was administered to arrest the heart. The aneurysm was carefully entered, revealing no thrombus inside the lumen.

**Figure 2:**
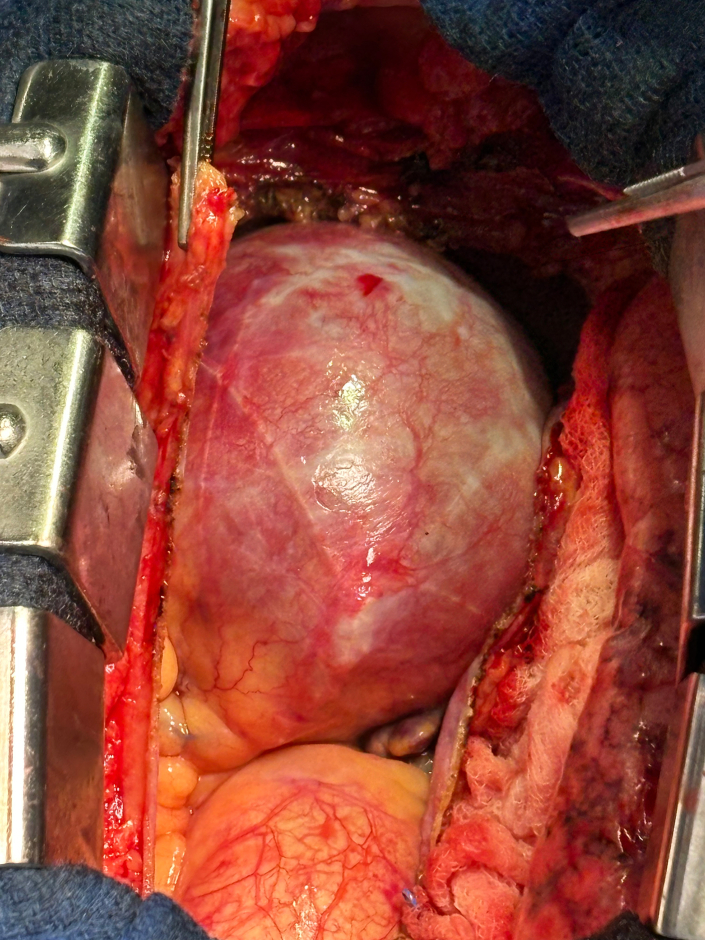
Large 8-cm right coronary artery aneurysm seen upon opening the chest and dissecting the pericardium.

The paroxysmal atrial fibrillation was treated with an EnCompass Clamp (Atricure) passed around all 4 pulmonary veins, which was fired 6 times in 3 sets of 2 ablations. Following the ablation, the atrial appendage was closed with a 45-mm V clip at its base. Cardioplegia was then administered to the proximal segment of the right coronary artery demonstrating the ostia, which was subsequently ligated within the aneurysm sac. The distal aspect of the right coronary artery was severely calcified and required ostial endarterectomy.

A reverse saphenous graft was then used to perform CABG to the distal segment of the right coronary artery located within the aneurysm sac. Both proximal veins grafts, from the OMA and right coronary artery, were successfully anastomosed to the aorta. The patient was weaned off cardiopulmonary bypass without difficulty.

Early postoperatively persistent chest tube output developed. After exploration, a small thymic bleed was identified and controlled. His subsequent postoperative course was uncomplicated. He recovered well and was discharged home on postoperative day 7. On his 1-month follow-up clinic visit he was stable and recovering from the operation well.

## Comment

CAAs are rare, with an incidence of 0.3% to 4.9% on angiography.^2^ Giant coronary aneurysms are even more rare, with an incidence of 0.02% to 0.2%.^4^ The incidence of a giant CAA ≥50 mm in diameter, as seen in this case, is on the lower end of the spectrum with a prevalence of 0.02%.^4^ A universally accepted definition of a giant CAA does not exist, but it has been defined in other literature as an aneurysm with a diameter >20 mm, 40 mm, 50 mm, or quadruple the reference vessel diameter.^4^ In this case, the CAA had a diameter of 8 cm and if left untreated, could have caused symptoms such as angina pectoris, sudden death, fistula formation, pericardial tamponade, compression of surrounding structures, or congestive heart failure.^4^

Although no standardized guidelines exist for the treatment of giant CAAs, management, whether medical, percutaneous intervention, or surgical, requires an individualized approach based on aneurysm morphology, clinical presentation, underlying etiology, and physician experience.^1^^,^^2^ Medical therapy focuses on antithrombotic regimens, often combining antiplatelet and anticoagulant agents, along with statins, angiotensin-converting enzyme inhibitors, and blood pressure control, particularly in patients with atherosclerosis, Kawasaki disease, or smaller aneurysms.^1^, ^2^, ^3^ Percutaneous coronary intervention, including conventional stent implantation and coil embolization, is generally reserved for smaller aneurysms or obstructive lesions.^1^^,^^2^ Additionally, other authors have reported successful outcomes using covered stents as a novel treatment option for smaller CAAs.^5^ Several surgical approaches have been described, including aneurysm reconstruction, resection, and ligation with concomitant CABG on-pump or off-pump.^1^, ^2^, ^3^, ^4^^,^^6^ In a series by Singh and colleagues,^6^ the predominant approach involved proximal ligation and plication of the aneurysm combined with CABG to restore perfusion. The choice between on-pump and off-pump procedure varied based on anatomy and surgical complexity. Graft selection included the left internal thoracic artery or saphenous vein, based on suitability. These cases illustrate that effective surgical management should be tailored based on individualized strategies.^6^

In our patient, the decision to proceed with surgical repair was driven by the aneurysm's large size, risk of rupture, and presence of significant calcification at the aneurysm's origin. Long-term follow-up with clinical surveillance and imaging will be essential to monitor for recurrence or new aneurysm formation. This report outlines a successful surgical strategy for managing a giant CAA, contributing to the broader understanding of treatment approaches in these rare and complex cases.

## Conflict of Interest Statement

The authors reported no conflicts of interest.

The *Journal* policy requires editors and reviewers to disclose conflicts of interest and to decline handling or reviewing manuscripts for which they may have a conflict of interest. The editors and reviewers of this article have no conflicts of interest.

## Supplementary Data

Video 1 Technique for the repair of a large 8 cm right coronary artery aneurysm. Video available at: https://www.jtcvs.org/article/S2666-2507(25)00280-9/fulltext.
