# Supplementary figures and images for: Giant 8-cm coronary artery aneurysm: Surgical management
Source: JTCVS Tech. 2025 Jul 18;33:139–41. doi: 10.1016/j.xjtc.2025.06.026 (PMC12529711; doi:10.1016/j.xjtc.2025.06.026)

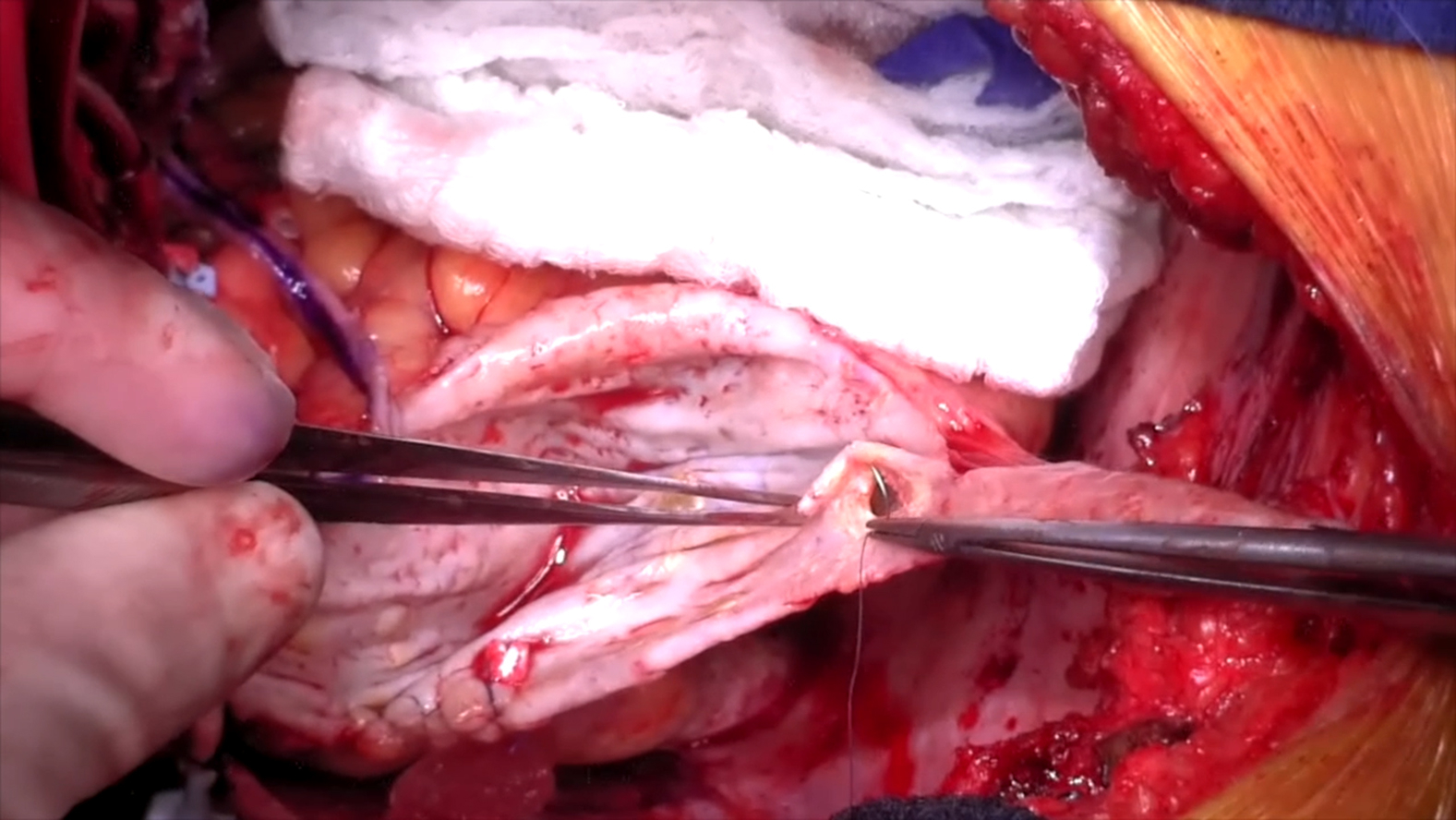

Supplement: Video 1 — Technique for the repair of a large 8 cm right coronary artery aneurysm. Video available at: https://www.jtcvs.org/article/S2666-2507(25)00280-9/fulltext. [file fx2.jpg]
